# *GABRB3* mutations: a new and emerging cause of early infantile epileptic encephalopathy

**DOI:** 10.1111/dmcn.12976

**Published:** 2015-12-09

**Authors:** Apostolos Papandreou, Amy McTague, Natalie Trump, Gautam Ambegaonkar, Adeline Ngoh, Esther Meyer, Richard H Scott, Manju A Kurian

**Affiliations:** ^1^ Genetics and Genomics Medicine UnitUCL Institute of Child HealthLondonUK; ^2^ Department of NeurologyGreat Ormond Street HospitalLondonUK; ^3^ Developmental Neurosciences ProgrammeUCL Institute of Child HealthLondonUK; ^4^ Department of Clinical GeneticsGreat Ormond Street HospitalLondonUK; ^5^ Department of Paediatric NeurologyAddenbrooke's HospitalCambridgeUK

## Abstract

The gamma‐aminobutyric acid type A receptor β3 gene (*GABRB3*) encodes the β3‐subunit of the gamma‐aminobutyric acid type A (GABA_A_) receptor, which mediates inhibitory signalling within the central nervous system. Recently, *GABRB3* mutations have been identified in a few patients with infantile spasms and Lennox–Gastaut syndrome. We report the clinical and electrographic features of a novel case of *GABRB3*‐related early‐onset epileptic encephalopathy. Our patient presented with neonatal hypotonia and feeding difficulties, then developed pharmacoresistant epileptic encephalopathy, characterized by multiple seizure types from 3 months of age. Electroencephalography demonstrated ictal generalized and interictal multifocal epileptiform abnormalities. Using a SureSelectXT custom multiple gene panel covering 48 early infantile epileptic encephalopathy/developmental delay genes, a novel de novo *GABRB3* heterozygous missense mutation, c.860C>T (p.Thr287Ile), was identified and confirmed on Sanger sequencing. *GABRB3* is an emerging cause of early‐onset epilepsy. Novel genetic technologies, such as whole‐exome/genome sequencing and multiple gene panels, will undoubtedly identify further cases, allowing more detailed electroclinical delineation of the *GABRB3*‐related genotypic and phenotypic spectra.

## Abstract 2

This article is commented on by Pearl on pages 330–331 of this issue.

**Abbreviations**
GABA_A_: Gamma‐aminobutyric acid type A
*GABRB3*: Gamma‐aminobutyric acid type A receptor β3 gene

**What this paper adds**

- A report of a novel *GABRB3* mutation associated with early infantile epileptic encephalopathy.
- A detailed description of the clinical and electrographic features of the case.
- A review of the few other reported cases of *GABRB3*‐related epileptic encephalopathy.
- A discussion of this gene's association with other neurodevelopmental disorder phenotypes.
- Increased clinical awareness of the role of multiple gene panels in the diagnosis of early infantile epilepsy.

Gamma‐aminobutyric acid type A (GABA_A_) receptors are ligand‐gated chloride channels that act as the primary mediators of fast inhibitory synaptic transmission in the central nervous system. They belong to the Cys‐loop superfamily, and are formed by pentameric assemblies of different subunit subtypes: α1–α6, β1–β3, γ1–γ3, δ, ε, π, θ, and ρ1–ρ3.^1^ Most GABA_A_ receptors contain two α‐subunits, two β‐subunits, and another, most commonly a γ‐subunit.^2^, ^3^ These subunits have four transmembrane domains, of which the second transmembrane domain forms a central ion pore with the other four subunits^4^ (Fig. 1a). When GABA, the physiological ligand of GABA_A_ receptors binds to the receptor, the ion pore opens, facilitating chloride influx or efflux. The direction of chloride flux depends on intracellular chloride concentration, regulated by the potassium‐chloride KCC2 and sodium–potassium–chloride NKCC1 co‐transporters. In developing brains, the predominance of NKCC1 leads to increased intracellular chloride, resulting in GABA‐mediated chloride efflux which depolarizes the cell membrane, thus leading to neuronal excitation.^5^ Mature neuronal cell bodies have low intracellular concentrations of chloride due to high levels of KCC2 expression, leading to GABA‐mediated chloride influx that hyperpolarizes the cell membrane leading to neuronal inhibition.^6^

**Figure 1:**
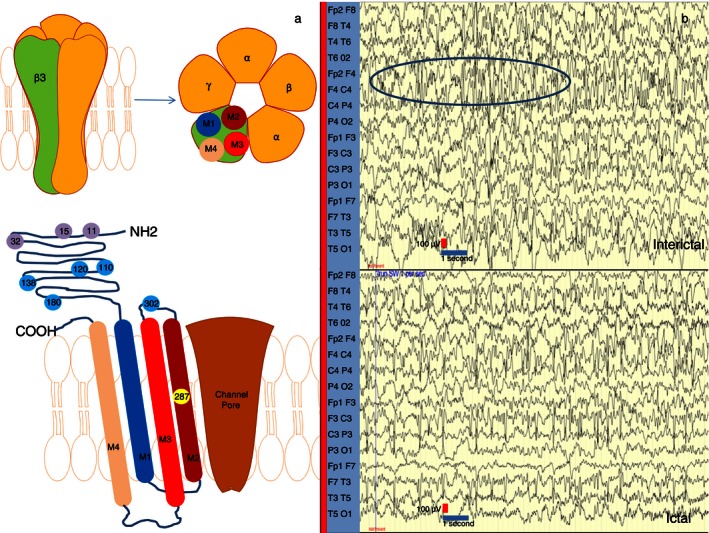
(a) Top: Schematic diagram of the gamma‐aminobutyric acid type A (GABA_A_) receptor and the gamma‐aminobutyric acid type A receptor β3 (*GABRB3*) subunit (depicted on the left). Most physiological heteromeric GABA_A_ receptors are thought to include two α‐, two β‐, and one other (most frequently a γ‐) subunits. Bottom: Each β3‐subunit consists of an extracellular domain, an α‐helical M1–M4 transmembrane bundle, and an M3–M4 intracellular loop. The M2 segments line the ion channel pore that tapers as it traverses towards the intracellular side of the membrane. The de novo heterozygous mutation in *GABRB3* in our proband is located in amino acid position 287. Thr287 is located in the M2 segment. Previously reported mutations in patients with epileptic encephalopathies are located in amino acid positions 110, 120, 138, 180, and 302. Polymorphisms and mutations implicated in childhood absence epilepsy are in positions 11, 15, and 32, clustered closer to the N terminus. All above mutations and variants are depicted as circles with corresponding numbers. (b) Interictal electroencephalogram (EEG) recording is seen above, showing high‐amplitude multifocal discharges, particularly in the right anterior region (circle). Ictal EEG is depicted below, demonstrating generalized fast activity, more prominent over the frontal regions.

Given the central inhibitory role of GABA_A_ receptors, it is not surprising that, to date, several genetic epilepsy syndromes have been associated with variants in GABA_A_ receptor subunit genes including *GABRA1*,* GABRB3*,* GABRD*, and *GABRG2*.^7^ *GABRB3*, located on chromosome 15q11.2‐q12, encodes the β3‐subunit of the GABA_A_ receptor. Reduced *GABRB3* expression has been postulated in the pathogenesis of absence seizures, abnormal sensory processing, and other neurodevelopmental disorder phenotypes such as Angelman syndrome, autism spectrum disorders, and intellectual disability.^8^, ^9^, ^10^ Furthermore, single nucleotide polymorphisms and missense mutations in *GABRB3* have previously been implicated in childhood absence epilepsy.^11^, ^12^

In 2013, *GABRB3* mutations were identified in patients with infantile spasms and Lennox–Gastaut syndrome.^13^ To date, few cases of *GABRB3* epileptic encephalopathy have been described in the literature. We report a case of early‐onset epilepsy with a de novo *GABRB3* mutation identified on a diagnostic multiple gene panel, and delineate the electroclinical phenotype in our patient.

## Case Report

A male, first‐born to unrelated white parents, was delivered after induction of labour, by forceps delivery at 42 weeks' gestation. The antenatal period was unremarkable and he was born in good condition. His maternal grandfather reported having seizures in childhood provoked by startling, but these resolved spontaneously and there were no further neurodevelopmental concerns. Family history was otherwise non‐contributory. The patient presented with neonatal hypotonia, feeding difficulties, and failure to thrive in early infancy. At 3 months of age, he developed clusters of short seizures characterized by eye deviation, eyelid flickering, tonic arm extension, and back arching. Ictal electroencephalography (EEG) revealed generalized fast activity, more prominent over the frontal regions, and interictal recordings showed high‐amplitude delta/theta multifocal (but predominantly right anterior) discharges (Fig. 1b). Initially, complete cessation of seizures was achieved in response to therapeutic doses of the GABA transaminase inhibitor vigabatrin. However, severe hypotonia, sedation, and respiratory difficulties ensued, hence the patient was weaned off vigabatrin and it was discontinued. Seizures recurred at 5months of age, and, despite multiple antiepileptic therapies (initially carbamazepine 14 mg/kg/day; then levetiracetam 40 mg/kg/day, topiramate 4 mg/kg/day, and sodium valproate 25 mg/kg/day in combination; and finally ketogenic diet with levetiracetam up to 50 mg/kg/day), he continued to have ongoing intractable epilepsy. At the time of last review, age 3 years 2 months, he had 10 to 20 epileptic seizures per day. Seizure semiology was varied, with episodic behavioural arrest, focal motor events, myoclonic jerks, and brief tonic seizures with lower‐limb extension. Parents also reported frequent paroxysms of laughter of undetermined origin, but these episodes have not been captured on EEG.

Over time, he developed severe global developmental delay with signs of slow progress but no obvious evidence of regression. At 3 years 2 months, head circumference plotted on the 0.4th centile. A few subtle dysmorphic features were evident, including mild prominence of his forehead with long eyelashes, tented mouth appearance, high‐arched palate, and bilateral undescended testes. There was also marked axial hypotonia and severe head lag. An overall paucity of limb movements was observed, although some antigravity movements were evident. Peripheral tone was low/normal, deep tendon reflexes were brisk throughout, and plantar responses up‐going bilaterally. There was no evidence of ankle clonus. He could make some vocalizations and was feeding orally with only occasional drooling, and no episodes of choking or swallowing difficulties. Extensive neurometabolic investigations were essentially unremarkable (Table SI, online supporting information), including a normal MRI brain (age 14 mo) and comparative genomic hybridization microarray analysis for genomic copy number variants.

## Molecular Genetic Investigation

Further diagnostic testing was undertaken using a multiple gene panel covering 48 genes (Table SII, online supporting information) causing early infantile epileptic encephalopathy. A custom SureSelect library was created (Agilent's SureDesign tool, https://earray.chem.agilent.com/suredesign/). Libraries were made following the SureSelectXT Custom Capture protocol (Agilent Technologies, Santa Clara, CA, USA) and sequenced in‐house on an Illumina MiSeq. Of targeted regions (target genes and their intron–exon boundaries), 99.7% were covered at ≥30×. A single heterozygous missense mutation of *GABRB3* (c.860C>T, p.Thr287Ile) was identified (Fig. 1a). Subsequent Sanger sequencing of both proband and parents confirmed these findings, establishing that the mutation was absent in the parents and likely to have occurred de novo (Fig. S1, online supporting information). The variant identified is classified as ‘likely pathogenic’ according to the American College of Medical Genetics/Genomics Standards and Guidelines,^14^ which infers greater than 90% certainty of being disease‐causing. This variant occurred de novo, providing strong evidence of pathogenicity. It was absent in control population databases including ExAC (http://exac.broadinstitute.org/), 1000 Genomes (http://browser.1000genomes.org/index.html), and Exome Variant Server (http://evs.gs.washington.edu/EVS/) (moderate evidence). Furthermore, the amino acid change occurs in a transmembrane domain lining the ion channel pore^4^ (moderate evidence). In silico analysis provided further evidence of pathogenicity, with Polyphen2 (score 1.000, sensitivity 0.00, specificity 1.00), SIFT (score 0.000), and PROVEAN (score −5.65) predicting the variant to be probably damaging, damaging, and deleterious, respectively.

## Discussion

We report a novel mutation in *GABRB3* in a patient with severe intractable early infantile epileptic encephalopathy. Our report highlights the recent finding that mutations in *GABRB3* are an emerging cause of early‐onset epilepsy syndromes.

To date, five other cases of *GABRB3* early infantile epileptic encephalopathy have been reported,^10^, ^13^ to our knowledge (Table 1). We postulate that there are several features common in all these cases, including: (1) seizure onset in infancy, <10months of age, (2) multiple seizure types including infantile spasms, (3) variable EEG abnormalities, and (4) associated comorbidities such as neurodevelopmental delay, attention‐deficit–hyperactivity disorder, autistic features, and intellectual disability. Similar to previous reported cases, our patient manifested features of a non‐specific early infantile epileptic encephalopathy disorder, with multiple seizure types and significant delay in development.

**Table 1:** Reported patients with early‐onset epileptic encephalopathy due to *GABRB3* mutations

Mutation	De novo/inherited	Age at onset (mo)	Seizure semiology	EEG	Seizure evolution and treatment response if reported	Other features	Intellectual disability	References
c.328A>G; p.Asn110Asp	De novo	5	Infantile spasms Myoclonic seizures	Hypsarrhythmia	No follow‐up data	None reported	None at presentation; no follow‐up data	Allen et al.^13^
c.358G>A; p.Asn120Asp	De novo	10	Infantile spasms	Generalized 2Hz bursts	Lennox–Gastaut syndrome	ADHD Impulsivity	Severe	Allen et al.^13^
c.413_415dupACC; p.Asn138_Arg139insHis	De novo	2	Myoclonic Focal seizures Atonic head nods	Multifocal/modified burst suppression	Responsive to levetiracetam and topiramate	‘Mild’ autistic features	Severe	Hamdan et al.^10^
c.539A>G; p.Glu180Gly	De novo	10	Infantile spasms	Generalized 2Hz bursts	Lennox–Gastaut syndrome	ADHD Impulsivity Sleeping difficulties	Severe	Allen et al.^13^
c.905A>G; p.Tyr302Cys	De novo	10	Focal dyscognitive seizures Behavioural arrests	Slow, some left temporal features	Lennox–Gastaut syndrome	None reported	Severe: 20 words at 4y	Allen et al.^13^
c.860C>T; p.Thr287Ile	De novo	3	Focal motor seizures Tonic seizures	Generalized fast activity. Interictal delta/theta multifocal discharges	Ongoing seizures with behavioural arrests, focal motor, myoclonic, tonic seizures	None currently	Severe	This paper

ADHD, attention‐deficit–hyperactivity disorder; EEG, electroencephalography.

Our patient harboured a mutation affecting a highly conserved protein domain located in the second transmembrane loop lining the ion channel (Fig. 1a). It is difficult to postulate what the exact pathogenic mechanism of this variant is without any in vitro or in vivo functional work; however, it is probably due to loss of function and haploinsufficiency. It is possible that the position of the amino acid change within the receptor pore could negatively impact on receptor function, for example by inhibiting the pore's opening and subsequent chloride flux. Other mechanisms including reduced GABRB3 protein expression and incorrect cellular trafficking of GABRB3 could also play a role. This is the first reported mutation affecting this region and, to date, most other reported *GABRB3* mutations causing epileptic encephalopathies target the extracellular protein domains, near the amino (N)′ terminus^13^ (Fig. 1a).

*GABRB3* has also been implicated in other neurodevelopmental disorders such as autism, childhood absence epilepsy, and Angelman syndrome. A single nucleotide polymorphism at the promoter region of *GABRB3* (T>C substitution in position −897, numbering with respect to the initiator methionine of exon 1a), which leads to reduction of the promoter's transcriptional ability, has been associated with childhood absence epilepsy in previous studies.^12^ Other researchers have reported that a rare *GABRB3* single nucleotide polymorphism (c.31C>T; p.Pro11Ser)^15^ and other variants (c.44C>T; p.Ser15Phe, and c.94G>A; p.Gly32Arg) (NM_021912.4) are linked with childhood absence epilepsy phenotypes associated with eyelid myoclonus and generalized tonic–clonic seizures.^6^, ^11^ Probands had typical generalized 3Hz spike and wave discharges during childhood, with clinical symptoms later remitting, without neurological sequelae. The identified mutations were also present in other family members who never had epileptic seizures, suggesting variable penetrance. The same mutations were not found in 630 healthy ethnically and sex‐matched comparison individuals in one study. However, p.Pro11Ser was later described in an asymptomatic individual, suggesting it may be a rare single nucleotide polymorphism.^6^, ^15^ Interestingly, genetic variants implicated in childhood absence epilepsy are clustered much closer to the N‐terminal domain of the protein than those identified in *GABRB3* early infantile epileptic encephalopathy (Fig. 1a).

In Angelman syndrome, deletions encompassing *GABRB3* have been reported.^16^ Mice with *Gabrb3* knockout have epilepsy and other abnormalities that show some similarities to patients with Angelman syndrome.^17^ The extent to which GABRB3 accounts for the clinical phenotype of Angelman syndrome is currently unclear. Other genes including *GABRG3* and *GABRA5* are also often contained within deletions causing Angelman syndrome.^16^ The more severe phenotype associated with such deletions might indicate an additive role for these genes in disease pathogenesis. EEG recordings in Angelman syndrome often demonstrate specific rhythmic patterns that are less prominent in patients with deletions encompassing *GABRB3*. Indeed, no such rhythmicity was evident in our participant.

At present, there are too few reported cases of *GABRB3*‐related epilepsy for clear phenotype–genotype correlations or comparisons with Angelman syndrome due to chromosome deletions with *GABRB3* haploinsufficiency. It is currently unknown whether mutation type and location affect clinical disease presentation, or whether other environmental or epigenetic factors may play a role. Increasing availability of novel genetic technologies, such as multiple gene panels and whole‐exome sequencing will undoubtedly accelerate the identification of further cases, allowing more detailed delineation of the spectrum of *GABRB3*‐related disorders.

Recent studies suggest that *GABRB3* mutations cause attenuated chloride currents and channel activity impairment through subunit hyperglycosylation.^11^ The mechanisms through which *GABRR3* mutations affect neuronal networks giving rise to epilepsy and neurodevelopmental delay are yet to be fully elucidated. Further research is needed, not only to understand the underlying disease pathogenesis but also to identify novel therapies, which may also have wider implications for GABA_A_ modulation in other neurological disorders.

## Supporting information


**Figure S1:** Top panel: De novo heterozygous mutation in *GABRB3*, c.860C>T (p.Thr287Ile) detected by panel and confirmed by Sanger sequencing (red rectangle).

**Table SI:** Neurometabolic investigations undertaken in patient.
**Table SII:** Epilepsy and severe delay gene panel result.
