# Supplementary material for: GABRB3 mutations: a new and emerging cause of early infantile epileptic encephalopathy
Source: Dev Med Child Neurol. 2015 Dec 9;58(4):416–20. doi: 10.1111/dmcn.12976 (PMC4864756; doi:10.1111/dmcn.12976)
Supplement: Supplementary file 1 — Figure S1: Top panel: De novo heterozygous mutation in GABRB3, c.860C>T (p.Thr287Ile) detected by panel and confirmed by Sanger sequencing (red rectangle). [file DMCN-58-416-s001.pdf]

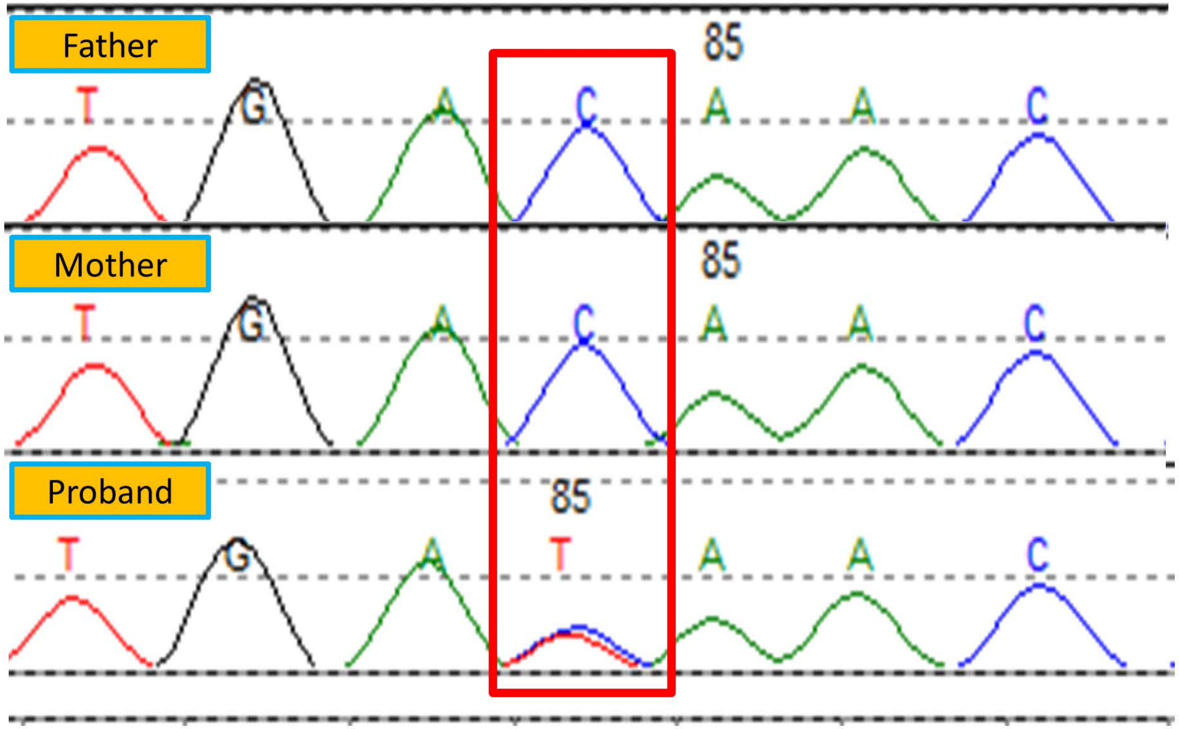

H.sapiens	263	VSFWINYDASAARVALGITTVLTMTTINTHLRETLPKIPYVKAIDMYLMG	312
P.troglodytes	263	VSFWINYDASAARVALGITTVLTMTTINTHLRETLPKIPYVKAIDMYLMG	312
M.mulatta	263	VSFWINYDASAARVALGITTVLTMTTINTHLRETLPKIPYVKAIDMYLMG	312
C.lupus	241	VSFWINYDASAARVALGITTVLTMTTINTHLRETLPKIPYVKAIDMYLMG	290
B.taurus	263	VSFWINYDASAARVALGITTVLTMTTINTHLRETLPKIPYVKAIDMYLMG	312
M.musculus	263	VSFWINYDASAARVALGITTVLTMTTINTHLRETLPKIPYVKAIDMYLMG	312
R.norvegicus	263	VSFWINYDASAARVALGITTVLTMTTINTHLRETLPKIPYVKAIDMYLMG	312
G.gallus	263	VSFWINYDASAARVALGITTVLTMTTINTHLRETLPKIPYVKAIDMYLMG	312
D.rerio	263	VSFWINYDASAARVALGITTVLTMTTINTHLRETLPKIPYVKAIDMYLMG	312
D.melanogaster	276	VSFWINHEATSARVALGITTVLTMTTISTGVRSSLPRISYVKAIDIYLVM	325
A.gambiae	254	VSFWINHEATSARVALGITTVLTMTTISTGVRSSLPRISYVKAIDIYLVM	303
C.elegans	281	VSFWINHEATSARVALGITTVLTMTTISTGVRQSLPRISYVKSIDIYLVM	330
X.tropicalis	263	VSFWINYDASAARVALGITTVLTMTTINTHLRETLPKIPYVKAIDMYLMG	312
