# Supplementary material for: GABRB3 mutations: a new and emerging cause of early infantile epileptic encephalopathy
Source: Dev Med Child Neurol. 2015 Dec 9;58(4):416–20. doi: 10.1111/dmcn.12976 (PMC4864756; doi:10.1111/dmcn.12976)
Supplement: Supplementary file 2 — Table SI: Neurometabolic investigations undertaken in patient. Table SII: Epilepsy and severe delay gene panel result. [file DMCN-58-416-s002.docx]

**Table SI:** Neurometabolic investigations undertaken in patient

|  | **Investigations** | **Results** |
| --- | --- | --- |
| **Blood** |  |  |
| **Biochemistry** | U&Es, LFTs, bone profile, lactate, glucose, ammonia, TFTs, CK, copper, urate, biotinidase, transferrin isoelectric focusing, amino acids, carnitine, acylcarnitine profiles, VLCFA, glycosylation studies, white cell enzymes, cholesterol, triglycerides | Normal |
| **Genetics** | SMN genes, Prader–Willi, myotonic dystrophy, POLG, karyotype, CGH microarray | Normal |
| **Urine** |  |  |
|  | Organic acids, sulphite, amino acids, a-AASA, oligosaccharides and GAGs, GAMT | Organic acids: mild increase in glycerate, possibly contamination, otherwise normal |
| **CSF** |  |  |
|  | B6 metabolites, folate and monoamine neurotransmitters, glucose, lactate, CSF to plasma glucose ratio, amino acids | Normal |
| **Muscle and skin biopsy** |  |  |
|  | Histology, electron microscopy and respiratory chain enzymes | Normal |
| **Imaging** |  |  |
|  | MRI brain | Normal |
| **Electrophysiology** |  |  |
|  | EEG | Interictal delta/ theta multifocal epileptiform discharges over both hemispheres, especially persistent over the right anterior region. Ictal generalised amplitude reduction and diffuse 14-16Hz fast activity (more prominent frontally) |
|  | EMG/ Nerve conduction studies | Normal |

a-AASA, alpha-aminoadipic semialdehyde; CGH, compatative genomic hybridization; CK, creatine kinase; GAGs, glucosaminoglycans; GAMT, guanidinoacetate methyltransferase; LFTs, liver function tests; POLG, polymerase gamma; SMN, survival of motor neuron (Spinal Muscular Atrophy); TFTs, thyroid function tests; U&Es, urea and electrolytes; VLCFA, very long chain fatty acids.

**Table SII:** Epilepsy and severe delay gene panel result

| **Genes tested** | **Conclusions** |
| --- | --- |
| *ADSL, ARHGEF9, ARID1A, ARID1B, ARX, ATP1A3, ATRX, CDKL5, CHRNA2,CHRNA4,CHRNB2,CNTNAP2, EHMT1, FOXG1, GABRG2,KCNQ2, KCNT1, KIAA1279, LGI1,MAGI2, MDB5, MECP2, MEF2C, NRXN1,PCDH19,PLCB1, PNKP, POLG,PRRT2, SCN1A, SCN1B, SCN2A, SCN8A, SLC16A2, SLC25A22, SCL2A1, SLC9A6, SMARCA2, SMARCA4, SMARCB1, SMARCE1, SPTAN1, STXBP1, SYNGAP1, TCF4, UBE2A, UBE3A, ZEB2* | No mutation found |
| ***GABRB3:*** | c.860C>T heterozygote, *de novo*, novel mutation;  Consistent with a diagnosis of GABRB3-related epileptic encephalopathy |

Screening carried out using next generation sequencing (Agilent SureSelect + MiSeq). A minimum of 30x coverage was required to call a variant. 99.7% of the coding bases in the targeted genes were covered >30x. In-house validation attributed a minimum sensitivity of 97.5% (with 95% confidence) for regions covered >30x.
